# A qualitative study describing an occupation-focused framework enabling children’s survival and activity functioning after brain injury in Uganda

**DOI:** 10.4102/ajod.v15i0.2100

**Published:** 2026-10-26

**Authors:** Francis U. Ekwan, Pamela J. Gretschel, Amshuda Sonday

**Affiliations:** 1 Department of Health and Rehabilitation Sciences, Faculty of Medicine and Health Sciences, Stellenbosch University, Cape Town, South Africa; 2 Department of Health and Rehabilitation Sciences, Faculty of Health Sciences, University of Cape Town, Cape Town, South Africa

**Keywords:** occupation-focused, framework, survival, functioning, participation, children, brain injury

## Abstract

**Background:**

Enabling children’s survival and activity functioning after brain injury remains a challenge to caregivers and health professionals in Uganda. In response, an occupation-focused framework (OFF) was developed during a doctoral study aimed at co-creating a culturally relevant programme to enable participation for these children.

**Objectives:**

This study describes the OFF and how it enables children’s survival and activity functioning after brain injury in Uganda.

**Method:**

Using a participatory action research design, this study sampled three doctors, five nurses, three therapists and three caregivers as general participant group members, and eight children with brain injury as primary participants. Participants were selected from two therapy settings in Kampala-Wakiso, Uganda. The general participants’ interactions with the children were video-recorded, and group interviews and reflections audio recorded. Data were analysed thematically.

**Results:**

This study revealed that bridging basic needs and enablement is central to the OFF and is mediated by an ‘occupation scale’ that reflects child activity fitness in context. Three core sub-themes were generated: assessing child enablement contexts, supporting caregivers to attain psychological health and engaging the child in daily life activities.

**Conclusion:**

This study supports the use of an occupation scale as a foundational principle for enabling children’s survival and activity functioning after brain injury across contexts such as home, school and hospital with relevant resources.

**Contribution:**

The study contributes to the rehabilitation field by introducing a scale that fosters collaborative assessment and interventions, furthering the potential to enable children’s activity functioning and survival after brain injury in resource-scarce environments like Uganda.

## Introduction

Brain injury is becoming a global disease burden with impactful and devastating outcomes (Dewan et al. 2018). The devastating nature of brain injury is more severe in low- and middle-income countries (LMICs) (Dewan et al. 2018). For instance, in many LMICs, it’s harder to identify, implement and evaluate prevention and intervention programmes for children with brain injury because there are no standards, data to inform decisions and limited human resource capacity to manage complicated forms of brain injury (Maas et al. 2015; Nampijja et al. 2019; Pal et al. 2016).

Brain injury refers to neural and non-neural cell damage in the brain leading to cell death or cellular dysfunction, resulting from external trauma such as a fall or from internal factors such as stroke (Pekna & Pekny 2012). Literature sources use specific descriptors – traumatic brain injury (TBI) or non-traumatic brain injury (nTBI), reflecting its causation. Traumatic brain injury happens when a sudden, external, physical assault damages the brain (Faul & Coronado 2015). Non-traumatic brain injury happens when internal factors such as hypoxia or anoxia, toxin, stroke, tumour, meningitis, cerebral oedema, hydrocephalus, encephalitis and aneurysm damage the brain (Stevens & Sutter 2013). Evidence shows that brain injury accounts for nearly one half of all trauma deaths and more than 10% of total global disability-adjusted life years (Punchak et al. 2018). Whereas the incidence of brain injury among children is thought to be higher in different African countries, the daily life occupation realities for children with brain injuries remain that of dependent participation (Idro 2015).

Studies on the therapeutic powers of occupation (Kielhofner 2008; Townsend & Polatajko 2007) support the belief that children with brain injury may be enabled to participate in diverse contexts using an appropriate framework grounded in a philosophical understanding of child’s occupation within the guiding principles of culture, disability, health and participation. These theories motivated the researcher’s efforts to invite diverse participants’ perspectives to co-create an occupation-focused framework (OFF) with an aim to enable children’s survival and activity functioning after brain injury in Uganda. The framework was informed by child-centred, occupation-focused and contextually relevant perspectives. This framework challenges previous strategies used by ABAaNA study (Nampijja et al. 2019) and Akwenda study (Asige et al. 2025; Saloojee et al. 2021), these studies, although designed for Ugandan children with brain injury condition, were caregiver-centred, child-body-activity-focused and relied on interventional ideologies imported into Uganda.

Most brain injury cases in Uganda result from head trauma affecting both motor and cognitive abilities with resultant long-term disability (Idro 2015; Kumar & Mahapatra 2009). Children of low socioeconomic status are most affected by brain injury because of their exposure to risky occupations such as street hawking and manning roadside shops (Udoh & Adeyemo 2013). Although children have better survival rates than adults with brain injury, the long-term consequences are often more devastating in children because their developmental potential could be hampered by the injury (Case-Smith & O’Brien 2014).

Based on the age factor, evidence shows that a child’s brain injury relates to health status (Okumura et al. 2013), access to care (Lotstein et al. 2005), functioning in daily activities (Donkervoort et al. 2008) and funding or insurance coverage (Kuo et al. 2014). In addition, age also relates to the nature of brain injury (Chevignard et al. 2010; McKinlay et al. 2016), with impactful and devastating consequences such as severe individual and family disruption and lifelong disability (Dewan et al. 2018). These impactful and devastating consequences require an OFF that must be culturally relevant in diverse contexts (Kokorelias et al. 2019; Kuo et al. 2012).

The Government of Uganda, *Persons with Disabilities Act (2020)*, as with the United Nations (2015) Convention on the Rights of Persons with Disabilities, emphasises measures to ensure the full enjoyment by children with disabilities of all human rights, including protection, education, health, rehabilitation and life (Government of Uganda 2020; United Nations 2006). Borrowing a leaf from You and friends, Ugandan children with brain injury and many others with disabilities deserve the best through research, management of disabilities, communication, care and support (You et al. 2015). Although the *Persons with Disabilities Act* of Uganda could be perceived as fostering inclusion and non-discrimination of persons with disabilities (You et al. 2015), there are challenges and gaps when it comes to matters of caregiving for children with brain injury; for example, often it is the mothers who assume the caregiving role for children, while fathers frequently abandon the family when children become disabled (Imamura et al. 2026). Because mothers struggle to raise their children with disabilities alone, they experience distress and emotional struggles in their daily lives (Imamura et al. 2026). In a study by Imamura and friends, mothers described themselves as ‘not doing well’ (Imamura et al. 2026). Mothers not doing well, is inevitable in an environment still yet honoring traditional and cultural beliefs that deny children with disabilities their real family name by using names such as “Ajok” or “Ojok,” meaning, “that of the spirit”; in addition, exposing children with disabilities to hostile environment with limited family support including assistive technology (AT) access for community use and participation (Andrews et al. 2023). These challenges require an appropriate framework for interventions which ensure children with brain injury experience inclusion in diverse community contexts which support their activity functioning and survival in daily life. The framework must promote involvement of both the mothers and fathers as key contributors in the provision of care and support for children with brain injury. Similar to what Imamura and friends reported, other studies conducted in Uganda showed that mothers of children with disabilities often experience distress and emotional struggles in their daily lives mainly because fathers leave them behind and usually these mothers do not access focused psychosocial support in getting their children with disabilities to engage in valued occupation such as education and developmental activities (Namazzi et al. 2019, 2020).

Children’s engagement in valued occupation was the focus of this study that took place at the Kampala-Wakiso Home Therapy Centre connected to one of the Uganda’s national referral hospitals. Kampala is the capital city of Uganda (International Growth Centre 2021). Wakiso is one of the new cities in Uganda, encircling Kampala (International Growth Centre 2021). Although Uganda’s health care system is one of the most robust decentralised health systems in Africa, it is structured to attend to disease complexity (Nampala 2018), and no feasible OFF was in place to guide rehabilitation of children with brain injury at different health service delivery levels. Thus, the purpose of this study was to co-create a culturally relevant programme that can enable participation of children with brain injury in Uganda (CEBU). The specific objective of this article is to describe the OFF enabling children’s survival and activity functioning after brain injury in Uganda.

## Research methods and design

### Study setting

The study was conducted in a home therapy centre, established under the Mulago National Referral Hospital, Uganda. Mulago National Referral Hospital is in Kampala; it serves a population of over 45 million people and currently has a capacity of 1600 beds (Kampala Capital City Authority 2025); 900 beds in lower Mulago and 700 beds in upper Mulago. The most common indication for admission in Mulago National Referral Hospital has been TBI (84.5%) (Luggya et al. 2022). Mulago National Referral Hospital offers surgical and medical services for both children and adult populations, networking with other hospitals in the country.

### Study design

The research process followed a qualitative approach using participatory action research (PAR) design to explore multiple perspectives from a wide sample of participants (Kemmis, McTaggart & Nixon 2014), guided by a constructivist philosophical perspective (Charmaz 2016).

Constructivism emphasises a belief in multiple realities that are linked to the diverse experiences of individuals formed through different interpretations, meanings and intentions (ed. Crotty 2020). Using a constructivist perspective enabled the researcher to explore multiple realities from the participants’ interpretations or constructions of their experiences, considering experience similarities, interpretations and correctness (Jaccard & Jacoby 2010).

### Study participants

The study used purposive sampling to select 14 general participants group (GPG) members and eight children with brain injury as primary participants. The GPG members included four participant group categories: three doctors, three therapists, three carer-to-carer (C2C) caregivers and five nurses. Each participant group category nominated two of its members to contribute to the core participants group (CPG), tasked with approving the preliminary analysis findings and final analysis of the findings (De-la-Cueva-Ariza et al. 2018).

### Participant selection

Eight children were selected based on five criteria:

- Age and diagnosis – children aged 2–17 years old diagnosed with brain injury condition(s) included, and those below two and above 17 years old excluded.
- Gross Motor Function Classification (GMFCS) (Palisano et al. 2008) – children with GMFCS level two to five included and those with GMFCS level one excluded.
- Manual Ability Function Classification (MACS) (Eliasson et al. 2006) – children with MACS level two to five included and those with MACS level one excluded.
- Communication Function Classification System (CFCS) (Hidecker et al. 2011) – children with CFCS level one to four included and those with CFCS five excluded.
- Glasgow Coma Scale (GCS) (Teasdale & Jennett 1974) – children with GCS level nine to 15 included and those with GCS level three to eight excluded.

The characteristics of children with brain injury in the study are shown in Table 1.

**TABLE 1:** Characteristics of children with brain injury in the study (*n* = 8).

Pseudonyms	SINo	Gender	Age (years)	Schooling	Diagnosis	GMFCS	MACS	CFCS	GCS
Atin	PN1	F	5	X0	CP	4	4	3	15
Omwa	PN2	F	11	X0	Hydro	3	2	1	15
Dazo	PN3	F	5	X0	CP	4	4	4	12
Amol	PN4	F	2	X0	CP	4	3	4	12
Mera	PN5	M	8	X1	TBI	5	3	2	15
Omol	PN6	M	14	X0	Stroke	2	2	2	15
Abil	PN7	F	3	X0	TBI	5	4	4	9
Bade	PN8	M	4	X0	Stroke	3	3	4	12

SINo, study identification number; GMFCS, gross motor function classification system; MACS, manual ability classification system; CFCS, communication function classification system; GCS, glasgow coma scale; CP, cerebral palsy; Hydro, hydrocephalus; TBI, traumatic brain injury; X1, child attending school; X0, child not attending school.

Three caregivers were selected from a list of five caregivers trained as C2C caregivers at Mulago National Referral Hospital. A caregiver was selected if involved in the Kampala-Wakiso Brain Injury Home Therapy Center (KWB-HTC) monthly, aged between 25 and 65 years, fluent in English, Ugandan by nationality and committed to participating in the study for 5–6 months. The list of caregivers trained as C2C caregivers was obtained from the head Occupational Therapy Department (OTD), Mulago National Referral Hospital with administrative clearance from the Director, Mulago National Referral Hospital.

Three therapists were purposively selected to participate in the GPG from a list of 12 therapists (six occupational therapists, four physiotherapists and two speech-language therapists) obtained from Mulago National Referral Hospital. A therapist was selected if involved in the KWB-HTC, aged between 25 and 65 years, fluent in English, Ugandan by nationality, committed to participating in the study for 5–6 months and registered with the Allied Health Professionals’ Council of Uganda. The list of therapists was obtained from the head OTD-Mulago National Referral Hospital with administrative clearance from the Director, Mulago National Referral Hospital.

Five nurses were purposively selected to participate in the GPG from a list of 11 nurses (four Neurosurgery, four Paediatrics and three Psychiatry) working at Mulago National Referral Hospital in the 2021/2022 financial year. Five nurses were recruited (two more than other GPG members) because the practice of nurses in paediatrics differs from that of other health professionals in terms of deployment; nurses are usually deployed in specific duty-bound clinical units at Mulago National Referral Hospital. Other health professionals, like doctors and therapists usually are deployed to cover a wider area, such as the entire paediatric or specialised clinical areas covering both paediatrics and adults. For contextual relevance, the researcher chose to recruit five nurses to represent five clinical units where children with brain injury usually seek care at Mulago National Referral Hospital: neuro paediatrics – medical (one nurse), neuro paediatrics – surgical (one nurse), paediatric nutrition (one nurse), psychiatry (one nurse) and paediatric intensive care (one nurse). A nurse was selected if involved in the KWB-HTC, aged between 25 and 65 years, fluent in English, Ugandan by nationality, committed to participating in the study for 5–6 months and registered with the Uganda Nurses and Midwives Council. The list of nurses was obtained from respective unit heads with administrative clearance from the Director, Mulago National Referral Hospital.

Three doctors were selected purposively to participate in the GPG from a list of 10 doctors (four Neurosurgery, three Paediatrics and three Psychiatry) obtained from Mulago National Referral Hospital. A doctor was selected if involved in the KWB-HTC, aged between 25 and 65 years, fluent in English, Ugandan by nationality, committed to participating in the study for 5–6 months and registered with the Uganda Medical and Dental Practitioners Council. The list of doctors was obtained from respective unit heads with administrative clearance from the Director Mulago National Referral Hospital.

The characteristics of caregivers, therapists, nurses and doctors in the study are shown in Table 2.

**TABLE 2:** Characteristics of caregivers, therapists, nurses and doctors in the study (*n* = 14).

Pseudonyms	SINo	Gender	Age (years)	Title	Years of practice
Onit	D1	F	41	Neurosurgeon	05
Fiti	D2	F	30	Psychiatrist	02
Twil	D3	F	44	Paediatrics doctor	08
Ono	N1	F	62	Paediatrics nurse	32
Twi	N2	F	59	Paediatrics nurse	29
Throg	N3	F	47	Neurosurgical nurse	17
Fout	N4	F	40	Paediatrics nurse	14
Fivi	N5	F	48	Nutrition nurse	21
Onivo	T1	M	42	Physiotherapist	14
Twori	T2	M	28	Occupational therapist	07
Twiti	T3	F	35	Speech-language therapist	11
Onup	C1	F	61	C2C Caregiver	05
Twic	C2	F	29	C2C Caregiver	04
Logu	C3	F	40	C2C Caregiver	05

SINo, study identification number; F, female; M, male.

After selection, phone calls were made to the selected caregivers and therapists using contact details obtained from OTD-Mulago National Referral Hospital, followed by invitation letters physically delivered; nurses and doctors were contacted through their head of department at Mulago National Referral Hospital. No participant dropped out during the study.

### Data collection and analysis

The process shown in Figure 1, describes the development of the OFF for enabling children’s survival and activity functioning after brain injury in Uganda.

**FIGURE 1:**
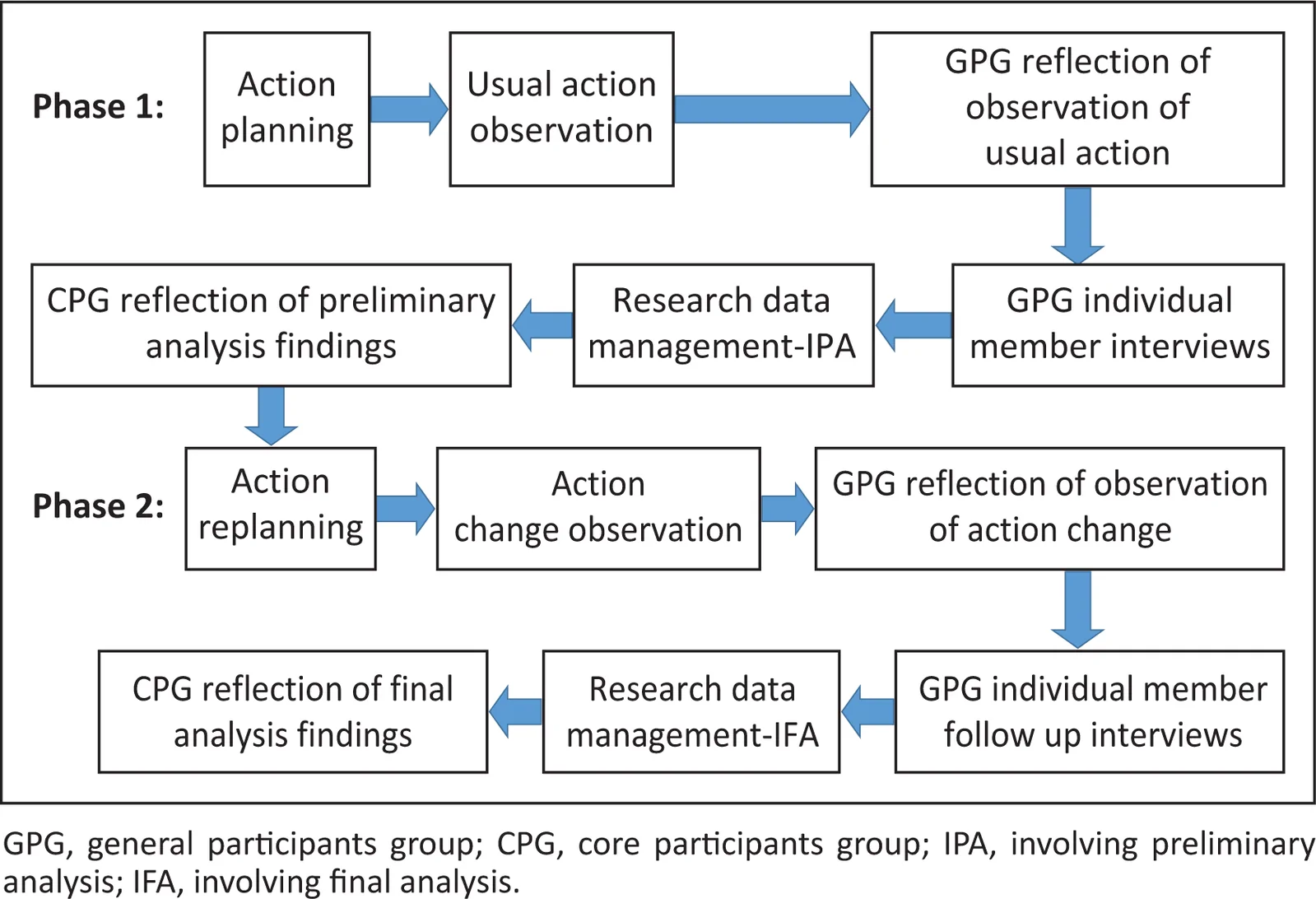
Flow chart describing the development of the occupation-focused framework for enabling children’s survival and activity functioning after brain injury in Uganda.

### Phase One

Six action steps were undertaken to establish the problems relating to the existing usual intervention provided for children with brain injury at KWB-HTC. This was informed by multi-level participants’ perspectives gained via self-observation, individual reflections, collective reflections and re-analysis of the significance of the usual interactional actions to reach the required change meant for phase two study (De-la-Cueva-Ariza et al. 2018).

#### Description of the six action steps

**Step 1: Action planning, January 2023:** This step involved preparing the participants for all aspects of data collection, clarifying their roles and highlighting human subject protection and precautions for coronavirus disease 2019 (COVID-19) and Ebola prevention.

**Step 2: Usual action observation, January 2023:** This step involved action observation during which time GPG members observed the children engaging in various activities. The process started with child one in action observation room one, while child two was being prepared in action observation room two. Each child spent 16 min per room; 10 min for action observation and 6 min for transition to the next room. Actions in rooms one to four included: undressing and body hygiene, exercise and assistive technology, play, medication and feeding, respectively.

Once child one reached action observation room four and child two reached action observation room one (exit rooms), the researcher invited child three to action observation room one and child four to action observation room two and the process continued with child one and two above until all eight children completed.

The GPG members observed that most children were irritated (for example, crying) during interactional actions.

**Step 3: General participants group reflection of observation of usual action, January 2023:** This step involved the participants reflecting on the action observations. The reflections featured valued self-reflection, discussions and proposals to the CPG for action change. Reflections followed a structured submission format: doctors, therapists and C2C caregivers each had to submit four submissions for collective reflection. The first three submissions were based on individual self-reflection of the usual interactional action observation of children with brain injury; and the last submission was based on the 5-min video recording by the research assistant; for each child observed in each action room. Nurses had six submissions for collective reflection. The first five submissions were based on individual self-reflection of the usual interactional action observation of children with brain injury, and the last submission was based on 5-min video recording by the research assistant; for each child observed in each action room.

**Step 4: General participants group individual member interview, January 2023:** Each GPG member was interviewed by a male researcher (PhD reg.) with over 10 years’ experience in paediatric neurological rehabilitation. The researcher used open-ended questions to encourage flexibility and open communication during interviews (Gallacher & Gallagher 2008).

**Step 5: Research data management involving preliminary analysis, January to March 2023:** Data consisted of 120 video clips, four audio recordings for GPG reflection, 14 audio recordings for GPG individual member interviews, 14 participation files for GPG members’ self-reflections, four sets of researcher’s field notes and one researcher’s journal.

Audio data were extracted from the video recorded and triangulated by matching action and audio of the interaction in the action-observation room. For example, a child’s cry was taken to mean discomfort and disapproval of the action. Audio recordings were then listened to familiarise self with the data content. Read the GPG member’s self-reflections, researcher’s field notes and journals. The researcher, together with one research assistant, completed the data coding.

Preliminary data analysis followed the six-step thematic analysis (Braun & Clarke 2006), generating four themes and 23 sub themes. Thematic analysis was used because it offered a more accessible and theoretically flexible approach for data analysis from different epistemological and ontological positions. Preliminary findings were presented, including a description of the coding tree to CPG members for further reflections.

**Step 6: Core participants group reflection of preliminary analysis findings, March 2023**: This step involved participation through review, data saturation discussion, and approval of GPG submission framed as six valued occupation plans for study phase two:

1. Using the Child Enablement Context Assessment (CECA) tool to assess the child’s functionality and enablement conditions; the usual intervention did not use CECA.
2. Counselling caregivers on brain injury to ease psychological pain; the usual intervention did not support constrained caregivers.
3. Sharing information among health professionals and caregivers about child health condition and activity fit; the usual intervention did not share information on child activity fit.
4. Using occupation-focused steps to enhance basic needs; the usual intervention did not use a step-by-step procedure but instead used unfocused goals.
5. Allocating 20 min for each basic need; the usual intervention allocated 16 min for each basic need.
6. Using five interactional action rooms; the usual interactional action used four action rooms.

### Phase two

Six action steps were undertaken to address the problems identified with the usual intervention for children with brain injury; this involved a multi-level participants’ engagement (De-la-Cueva-Ariza et al. 2018).

#### Description of the six action steps for study phase two

**Step 7: Action re-planning, April 2023:** This step involved seeking participants’ agreement with the decisions of the CPG on six valued occupations and assessing each of the eight children with brain injury using the CECA tool.

**Step 8: Action change observation, April 2023:** This step involved participation through action change and observation in a guided manner. Action change observation started with child one in action change observation room one, while child two was being prepared in action change observation room three. Each child spent 20 min per room; 15 min for action change observation and 5 min for transition to the next room. The action changes which occurred in room one to five were described as assistive technology-medicine use (AT7M7); oral-body hygiene (OH4BH10); posture-joint exercise (P4JE8); feeding on formulated food (FFF6); play-social relationships-class (P3S2C3).

Once child one reached action change observation room five and child two reached action change observation room two (exit rooms), the researcher invited child three to action change observation room one and child four to action change observation room three, and the process continued as with child one and two above until all eight children completed it.

The GPG members expressed satisfaction with the action changes.

Onup, said: ‘I am so happy…..with action changes, no child cried today’.

**Step 9: General participants group reflection of observation of action change, April 2023:** The reflections featured valued self-reflections, discussions and proposals to the CPG for final action change. The same structured submission format per GPG category earlier used for GPG reflection of action observation was also used for GPG reflection of action change observation.

**Step 10: General participants group individual member follow-up interview, April 2023:** Each GPG member attended a follow-up interview, averaging 1 h at Mulago National Referral Hospital (MNRH). The researcher member checked the first interview transcript, which generated additional data for discussion on the occupation-focused framework, including its consequences and limitations. Open-ended questions were used during the follow-up interview, and each GPG member was encouraged to maintain open communication (Gallacher & Gallagher 2008).

Step 11: Research data management involving final analysis, April to August 2023: This step followed similar analysis steps used during study phase one. Final analysis examined data in a relational manner. Data included, 120 video clips for study phase one and 150 video clips for study phase two; four audio recordings of GPG reflection in study phase one and four audio recordings of GPG reflection in study phase two; 14 audio recordings of GPG individual member interviews and 14 audio recordings of GPG individual member follow-up interviews; 14 participation files for self-reflections of the GPG members during individual interview; eight sets of researcher’s field notes, and one researcher’s reflective journal. In Table 3, this study presents the compiled data sources and data values for both study phases that led to the development of the OFF.

**TABLE 3:** Compiled data sources and data values for study phase one and phase two.

Data sources	Data values
Children with brain injury	Phase one: 120 video clips as additional evidence for the GPG reflections on usual occupation elements and changes required.
	Phase two: 150 video clips as additional evidence for the GPG reflections on new occupation elements and its occupation scale.
GPG doctors	Phase one: Two h’ audio record; group reflection (11 pages) and individual interview (24 pages) on usual occupation.
	Phase two: One h audio record; group reflection (6 pages) and individual follow-up interview (10 pages) on the new occupation elements and occupation scale descriptions.
GPG nurses	Phase one: Two h’ audio record; group reflection (12 pages) and individual interview (38 pages) on usual occupation.
	Phase two: One h 30 min audio record; group reflection (9 pages) and individual follow-up interview (28 pages) of the new occupation elements and occupation scale descriptions.
GPG therapists	Phase one: Two h’ audio record; group reflection (19 pages) and individual interview (42 pages) on usual occupation.
	Phase two: One h audio record; group reflection (8 pages) and individual follow-up interview (17 pages) of the new occupation elements and occupation scale descriptions.
C2C Caregivers	Phase one: Two h’ audio record; group reflection (14 pages) and individual interview (29 pages) on usual occupation.
	Phase two: One h audio record; group reflection (5 pages) and individual follow-up interview (18 pages) of the new occupation elements and occupation scale descriptions.
CPG (Drs; Nrs; Thers; Cgs)	Phase one: Review and approval of preliminary analysis findings represented as six valued occupation plans.
	Phase two: Review and approval of final analysis findings represented as the occupation-focused framework.

GPG, general participants group; CPG, core participants group; C2C, carer-to-carer; Drs, doctors; Nrs, nurses; Thrs, therapists; Cgs, caregivers.

Data analysis was done manually, following the six-step thematic analysis (Braun & Clarke 2006), as earlier described for preliminary analysis – study phase one. The researcher, together with one research assistant, did data coding. Final analysis of the findings was presented to CPG for final reflections on what would constitute the OFF for enabling participation of CEBU.

**Step 12: Core participants group reflection of final analysis findings, August 2023:** This was the 12th step. The final analysis findings were presented including a description of the coding tree, data saturation was discussed and approved by the CPG members. The approval included what this article presents as ‘occupation-focused framework for enabling children’s survival and activity functioning after brain injury in Uganda’.

### Scientific rigour: Trustworthiness

The GPG and CPG members discussed in detail each construct represented in the OFF with strict adherence to the research protocol; methods, processes and set question(s) to ensure trustworthiness (Coughlan, Cronin & Ryan 2007). To affirm credibility, the study discussed in detail each of the data elements contributing to the development of OFF for enabling children’s survival and activity functioning after brain injury in Uganda (Shenton 2004). The description was grounded on the 12-step research process demonstrating a fair and transparent research undertaking (Shenton 2004). In addition, a reflective researcher’s journal was used to track the research process; demonstrating confirmability (Shenton 2004). For transferability, the study has generated an occupation scale, a promising scale that can be cross-culturally adapted for use in other African contexts (Coughlan et al. 2007).

### Ethical considerations

Ethical approval was obtained from the Uganda National Council for Science and Technology, an accredited research governing body in Uganda (HS2509ES). This study was conducted in accordance with the World Medical Association (2024) Helsinki Declaration for research involving human subjects. Informed consent was received from all participants; assent was received from children aged 8 years old and above; parental consent was received for children below 8 years old and those above 8 years old with cognitive challenges that limited their ability to assent. Collected data was kept in a password-protected computer, accessible by the researcher only. Other research materials were kept in the research office, accessible by the researcher only. Two research assistants tasked with video recording in the action rooms signed a confidentiality agreement prior to being allowed to perform their task.

## Results

Based on the data, this study developed an OFF (Figure 2), for enabling children’s survival and activity functioning after brain injury.

**FIGURE 2:**
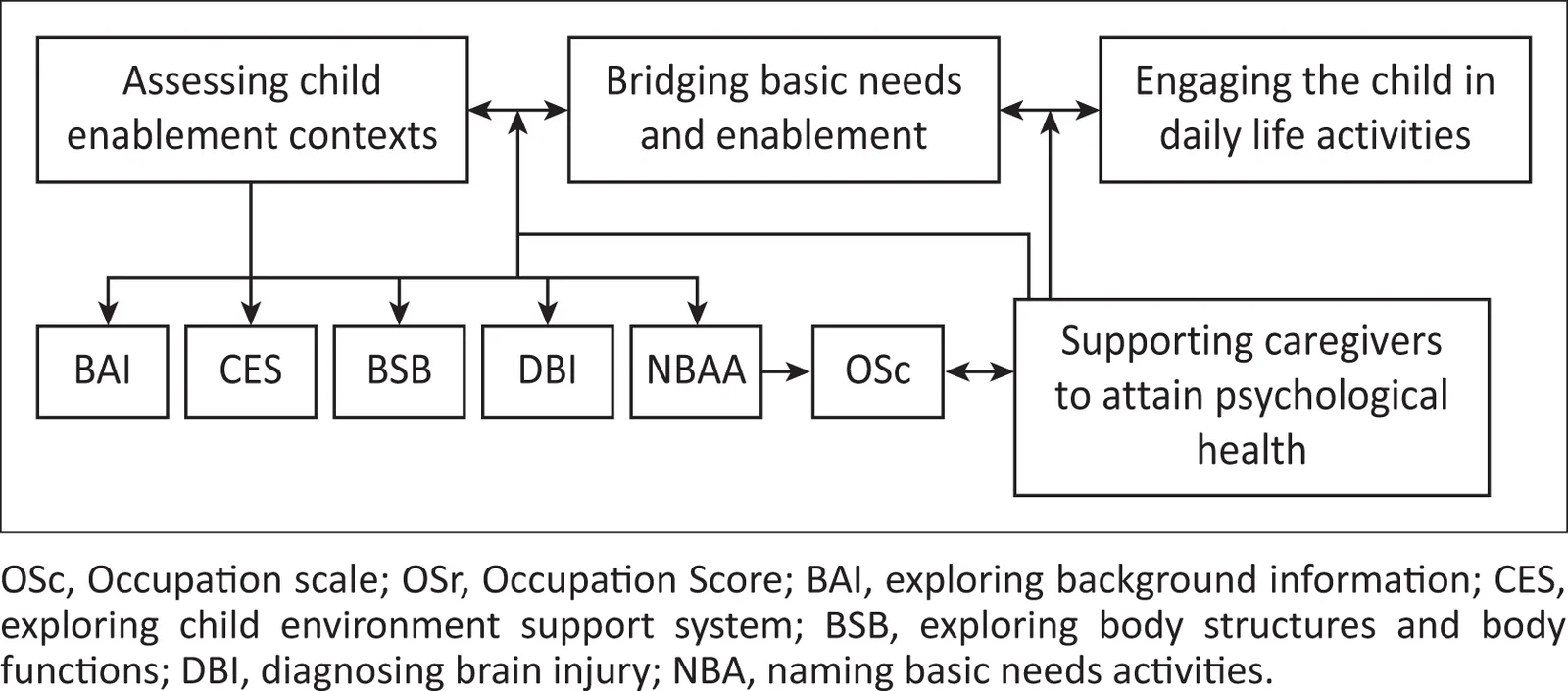
Occupation-focused framework enabling children’s survival and activity functioning after brain injury.

The process involved six steps: selection of statements, key words, coding, theming, conceptualising and conceptual framework development. The coding step generated 348 codes that were used to produce one final theme, three final sub-themes and their related concepts (Table 4).

**TABLE 4:** Theming and conceptualising the occupation-focused framework.

Final theme and sub-themes	Concepts for the occupation-focused framework
Theme: Bridging basic needs and enablement	Bridging basic needs and enablement in relation to assessing child enablement contexts, supporting caregivers to attain psychological health, engaging the child in daily life activities.
*Sub-theme 1:* Assessing child enablement contexts	Assessing child enablement contexts in relation to BAI, CES, BSB, DBI, NBA, occupation scale, bridging basic needs and enablement.
*Sub-theme 2:* Supporting caregivers to attain psychological health	Supporting caregivers to attain psychological health in relation to occupation scale, assessing child enablement contexts and engaging the child in daily life activities.
*Sub-theme 3:* Engaging the child in daily life activities	Engaging the child in daily life activities in relation to bridging basic needs and enablement.

BAI, exploring background information; CES, exploring child environment support system; BSB, exploring body structures and body functions; DBI, diagnosing brain injury; NBA, naming basic needs activities.

Each of these concepts were used to develop the OFF (Figure 2) that was approved by CPG members with only one minor modification to the occupation scale i.e. incorporating functional levels one, two, three, four and five to the occupation scale: +2, +1, 0, −1 and −2 (Table 5). This modification was done to enable the users to undertake a quantitative description of children’s functional capacity after brain injury. For example, a child with high functional capacity, maximum independence, could also be referred to as functional level five.

**TABLE 5:** Describing functional capacity for survival after brain injury.

Occupation scale	Description of functional capacity
OSc +2:	High functional capacity (Max. independent or level five)
OSc +1:	Middle-high functional capacity (near Max independent or level four)
OSc 0:	Middle functional capacity (moderate independent or level three)
OSc −1:	Low functional capacity (minimal independent or level two)
OSc −2:	Total functional incapacity (dependent or level one)

OSc, Occupation scale; Max, maximum.

### Bridging basic needs and enablement

Bridging basic needs and enablement was conceived as the central theme or concept for OFF. It applies the principle: ‘Child activity fitness in context’. Bridging basic needs and enablement relates to three sub-themes or concepts: assessing child enablement contexts, that is, culminates in occupation scale; supporting caregivers to attain psychological health, that is, links to occupation scale; engaging the child in daily life activities, that is, involves desirable actions.

Participants emphasised the relevance of appropriate help in enablement of children with brain injury, guided by the ‘child-fit’ principle.

Onivo, 42 years old physiotherapist emphasised that ‘only relevant help should be provided to the child’. In support, Twiti, 35 years old speech-language therapist added: ‘Providers of help should stick to the child’s cultural activity’. Referring to the child’s needs, Twori, 28 years old occupational therapist said: ‘The appropriateness of help depends on the child’s need and collaborative response’.

In this study, appropriate help and information sharing were seen as action changes in diverse cultural contexts involving a child with brain injury, health professional and caregiver. General participants group members emphasised that information sharing must consider the child’s health condition and activity fit.

Logu, 40 years old caregiver said: ‘Children’s body-activity functioning after brain injury determines the likely psychological health of caregivers and the survival of children themselves’. Fiti, 30 years old psychiatrist emphasised the need to support caregivers’ psychological health through what was described as ‘occupation scale’. General participants group members agreed with the cultural relevance of information sharing. Through shared reflections, the GPG-CPG members approved the use of ‘occupational scale’, linking it with context assessment and caregiver’s psychological health. Similarly, Twil, 44 years old paediatric doctor together with Omo, 62 years old paediatric nurse observed relevant action changes in children’s activity functioning within a 24-h time space; through further shared reflection, GPG-CPG members named it ‘occupation scale’ which formed part of the OFF. Onit, 41 years old neurosurgeon emphasised the value of the occupation scale, describing it as ‘an eye opener for helping children who are caught between life and death as a result of limited body-activity functioning’.

### Assessing child enablement contexts, supporting caregivers to attain psychological health and engaging the child in daily life activities

Assessing child enablement contexts was the starting point of the OFF (Figure 2). The GPG-CPG members approved eight contexts or processes that formed the occupation-focused framework: Exploring background information, exploring child environment support system, exploring body structures and body functions, diagnosing brain injury, naming basic needs activities and agreeing on occupation scale; upon occupation scale agreement, caregivers would experience the required psychological health to engage a child with brain injury in selected activities which have to fit the child’s body functioning and neuropsychological condition.

Onit, 41 years old neurosurgeon emphasised the need to assess and diagnose brain injury in children based on ‘neuropsychological examination’.

Conceptually, the GPG-CPG members described the occupation scale in terms of functional capacity for survival after brain injury (Table 5). Zero is the starting (neutral) point of survival. From the starting point, a child may be either enabled (go up), that is, positive scale or disabled (go down), that is, negative scale.

General participants group (GPG) members emphasized the importance of doing comprehensive assessment using CECA tool on first interaction between child-caregiver-health professional that includes baseline occupation scale for each ‘valued occupation’ (for example, feeding on formulated food, playing with other children and exercising body). Upon return, usually after 1 month of child-caregiver engagement, the same child would be assessed using the scale to reflect whether there was positive or negative or stagnation from the earlier scaled ‘valued occupation’ based on reported and observed child’s functional achievement or challenges.

Based on the capability ideology (Kakooza-Mwesige et al. 2018), the GPG-CPG members described the use of the occupation scale to rate the child’s functional capacity in terms of ‘dependent occupation (DO)’ or ‘independent occupation (IO)’.

Onivo, 42 years old physiotherapist said: ‘A child with brain injury is either capable or incapable’. If the child is capable, there is no need for help; if the child is incapable, there is a need for help. Twori, 28 years old occupational therapist affirmed: ‘Help for a child with brain injury reflects DO;’ whereas no help for a child with brain injury reflects ‘independent occupation’. Ono, 62 years old paediatric nurse described how health professionals and caregivers use occupation scale: ‘Occupation scale motivates health professionals and caregivers to do joint assessment of a child with brain injury’.

## Discussion

This study described the OFF co-created to promote children’s survival and activity functioning after brain injury in Uganda. Its main findings revealed that bridging basic needs and enablement is central to the OFF and is mediated by ‘occupation scale’ that reflects the child activity fitness in context. By generating three core sub-themes, that is, assessing child enablement contexts, supporting caregivers to attain psychological health and engaging the child in daily life activities, this study demonstrated how valued occupation can positively change children’s lives after brain injury. Other studies described valued occupation as ‘occupation in esteemed context’ (Kielhofner 2008; Townsend & Polatajko 2007). In this study, valued occupation constitutes positive occupation scale (+2 and +1) and neutral occupation scale (0) at functional capacity level. Positive occupation scale is what the study participants described as high functional capacity and middle-high functional capacity. On the other hand, participants described neutral occupation scale as middle functional capacity. For its occupation contextual meaningfulness, the study linked functional capacity to survival after brain injury.

Respecting the valuable contributions of all professionals, this study emphasises the situated nature of occupation in context and respect for context meaningfulness, as it’s the foundation upon which children’s engagement can be attained and which must be guided by using the CECA tool in addressing the neurological functional challenges described in this study by Onit, 41 years old neurosurgeon as ‘neuropsychological examination’. From a neuropsychological assessment point of view, rehabilitation plans and individual’s needs are central in ensuring meaningful intervention (Jarrahi et al. 2020), and guided by individual’s functionality – cognitive function, attention, memory, problem-solving, language, visuospatial skills and emotion (Tian et al. 2023). The current study focused on eight elements of valued occupation or processes derived from child-environment; the child’s engagement in valued occupation is powered by the caregiver’s psychological health. For example, this study found that without enhanced psychological health, most caregivers tend to use violent communication during their interactions with children; Abil, a 3-year-old child with traumatic brain injury, had a caregiver who said: ‘I am tired of you, for how long will I suffer with you, and if you don’t want to feed leave!’.

This study argues that without appropriate support for caregivers’ psychological health, children’s survival would be at risk because of their functional dependence on caregivers for basic needs such as feeding. Onivo, 42 years old physiotherapist, observed a child with brain injury as either capable i.e., independent of caregivers or incapable i.e., dependent on caregivers. Furthermore, Twori, 28 years old occupational therapist, argued that if a child with brain injury is dependent on caregivers, then such a child could require extensive help. Because of extensive help, this study proposes that caregivers’ psychological health can be supported by health professionals sharing information and guiding on the appropriate help that a specific child with brain injury would require based on the agreed occupation scale. Other studies (Bergström et al. 2015; Egan et al. 2014; Singam et al. 2015) support a belief that without valued occupation and relevant support to caregivers, several children with brain injury could die; others could suffer from activity functional loss, regress in life satisfaction and their caregivers could experience poor health outcomes as a consequence of stress from seeing and caring for their struggling children. This study asserted that every child with brain injury deserves to have their functional needs determined using an occupation scale which must be used collaboratively. Participants believed that addressing the contextual needs of children with brain injury requires collaboration between caregivers and health professionals in assessment and intervention. Collaboration is described in another study to reduce caregiver constraints (Dambi et al. 2017). Arguably, in the context of children with brain injury, collaboration would require the use of an appropriate functional classification system.

Several functional classification systems were used in this study: Gross Motor Function Classification (GMFCS) (Palisano et al. 2008), MACS (Eliasson et al. 2006) and CFCS (Hidecker et al. 2011). Although these classification systems supported the development of the OFF within the applicable principles of the occupation fit (Law et al. 1996), none of these classification systems would function as the OFF. It is important to note that the OFF was not developed as a classification system, but rather a theoretical foundation meant to guide functional rehabilitation of children with brain injury while maintaining close attention to valued occupation and functional capacity. A GCS (Teasdale & Jennett 1974) was also used in this study to guide selection of children’s participants. This study argues that the GCS and OFF differ in terms of purpose and components. Whereas the GCS assesses the neurological functioning of a patient with brain injury, focusing on consciousness – eye, verbal and motor responses (Teasdale & Jennett 1974), OFF guides the assessment of functional capacity for a patient (child with brain injury), focusing on activity functioning and survival. Therefore, the GCS strengthens the OFF (for example, the neuropsychological elements in the OFF include the three elements of the GCS mentioned above). Onit, a 41 years old neurosurgeon, was curious about understanding the neuropsychological challenges of children with brain injury using both GCS and OFF principles. Based on the shared reflection by Onit, other participants in the study supported inclusion of the three elements of the GCS while focusing on valued occupation and enablement choices.

This study, therefore, adds to the existing knowledge about valued occupation (Kielhofner 2008; Townsend & Polatajko 2007) and enablement (World Health Organization [WHO] 2007), citing occupation scale and caregivers’ psychological health support as the foundation for OFF enabling children’s survival and activity functioning after brain injury in Uganda. In this case, the occupation scale would expunge negative survival factors by addressing functional challenges experienced by children with brain injury, but in a collaborative manner to achieve occupation fit. Using the occupation scale is what Onivo, 42 years old physiotherapist, emphasised in relation to capacity ideation. This study, therefore, supports the use of occupation fit perspectives in applying occupation scale in context (Law et al. 1996).

By using the occupation fit perspectives (Law et al. 1996), this study assures children’s activity functioning and survival in contexts involving caregiving. In addition to using the occupation fit perspectives, the OFF also aligns with the World Health Organization International Classification of Functioning, Disability and Health – Children and Youth version (WHO-ICF-CY) (2007) based on the enablement and activity functioning ideology. However, the two frameworks (OFF and ICF) remain conceptually different: while OFF is grounded in child survival based on functional capability in daily life activities reflected as valued occupations in esteemed context, ICF is grounded in enablement as a complex interrelationship between body structures, activity functioning, participation and environmental factors (WHO 2007). Furthermore, the ICF does not include valued occupation in esteemed context as a culturally relevant way of enabling activity functioning and participation but instead the ICF places greater emphasis on environmental factors which this study found limiting in terms of occupation fitness and given the complexity of how brain injury presents in children. This study’s use of valued occupation and esteemed context is arguably a new addition to the WHO-ICF-CY if we must maintain its relevance in addressing the functional challenges that threaten survival of children with brain injury in diverse contexts. In this case, esteemed context refers to home, school and hospital with relevant resources to address the functional or rehabilitation needs of children with brain injury. Further still, this study goes on to assert that the occupation scale supports caregivers’ attainment of psychological health through a process described by Ono, 62 years old paediatric nurse as ‘joint assessment’. By joint assessment i.e. both the caregiver and health professional working collaboratively to complete the occupation scale for a specified child being assessed, this study is trying to demonstrate how relevant and inseparable the caregiver and a child with brain injury could be in the face of enablement and survival that the WHO-ICF-CY (2007) did not look into for responsive caregiving, functioning and participation.

Moving forward, this study recommends the use of occupation fit perspectives and occupation scale to represent valued occupation within the applicable principle of DO or IO. The two constructs form important guidelines for occupation engagement in esteemed contexts. As noted from this study, help for a child with brain injury means ‘dependent occupation;’ whereas no help means ‘independent occupation’. Therefore, the use of occupation scale is consistent with the capacity ideology that looks at children with brain injury as either functionally capable or incapable (Ekwan et al. 2023; Kakooza-Mwesige et al. 2018). As such, body-activity functioning remains the most daunting challenge for most Ugandan children with brain injury; negatively impacting on the caregivers’ psychological health. Occupation-focused framework developed following a 12-step participatory action research (PAR) process, therefore, uses the occupation scale as a core principle for meaningful actions aimed at delivering positive changes in children’s survival and activity functioning after brain injury in Uganda.

### Strengths and limitations

The study’s strengths and limitations are as follows:

- This study included doctors, nurses, therapists, caregivers and children with brain injury which allowed sharing of diverse views during the GPG and CPG discussions. It could have been a more balanced perspective if more opportunities were created for children’s voices by including a general participants group consisting of higher functioning children with brain injury capable of engaging in either verbal or non-verbal communication. Although this opportunity could be challenging given the complexities involved in terms of verbal expression after brain injury. However, a lack of children’s voices may limit future implementation of this study findings in practice and similar research undertakings where efforts are directed at collection of children’s voices.
- The limited functionality of children with brain injury could have restricted their full expression of verbal inputs. It is, however, hard to draw conclusions that fully represent children’s position where diverse views reflected upon are principally for health professionals and caregivers; children’s voices only featured through reflections on the video clips. This limitation could be addressed in similar future studies by facilitating children’s expression and voicing through art or using alternative augmentative communication (AAC) for children to engage and reflect on valued occupation in esteemed context. Furthermore, future research could explore how art or AAC enables expression of children’s voices in valued occupation contexts after brain injury.
- Future research needs to explore the usability of this framework across additional contexts such as refugee settlements.

## Conclusion

This article promotes the 12-step PAR process that generated valued occupations for CEBU. It supports the use of occupation scale (+2, +1, 0, −1, −2) as part of the OFF. Henceforth, introducing a scale that fosters collaborative assessment and interventions, ultimately enabling children’s activity functioning and survival after brain injury in resource-scarce environments like Uganda. However, the use of this new scale would require further research, including cross-cultural adaptation and validation of the psychometric properties of occupation scale in the Uganda cultures.

From a methodological standpoint, reviewed literature showed that no study has ever used the 12-step PAR process in coming up with a culturally appropriate intervention for children with brain injury anywhere globally. By far, this could serve as an important starting point in researching culturally relevant occupation-focused interventions for children with brain injury in esteemed contexts such as home, school and hospital with relevant resources.

The existence of caregivers’ experience in caring for children with disabilities and specifically brain injury is well addressed in the literature. However, little or nothing is currently available on the collaborative involvement of caregivers, health professionals and relevant community members in the occupation engagement of children with brain injury based on occupation scale. This study, therefore, recommends occupation engagement for children with brain injury guided by an OFF that includes occupation scale. By unlocking this new discovery, there are increased possibilities for inclusive and collaborative care and support for children with brain injury in a resource-scarce setting like Uganda, traditionally known for disjointed and uncoordinated service provisions to children with brain injury and other disabilities.

In addressing the current knowledge gap on inclusion of children’s voices in the design and implementation of occupation-focused interventions, further research is needed to explore children’s use of art or AAC to express voices in valued occupation.
